# The Doctor's Domestic Intellect

**Published:** 1890-03-29

**Authors:** 


### The Hospital

A Weekly Journal of

Science, Medicine, Nursing, and Philanthropy.

*For Hospitals, Asylums, Medical Practitioners, Students, Nurses, Families, Parishes, Congregations, and the Charitable Public.*

Vol. VII.—No. 183.] [March 29, 1890.

### The Doctor's Domestic Intellect.

"History is philosophy teaching by examples." So said Bolingbroke, quoting from Dionysius of Halicarnassus. As the philosophy of individuals and nations works itself out and becomes objective in their history, so, conversely, the daily occupations of the average man work themselves into his brain and consciousness and become his philosophy and himself. A man is what he does, exactly as a man does what he is. The intellect of the average doctor is of the domestic order. The nature of his daily avocations makes this, to some extent, inevitable. His business lies in the home; women and children are his most constant care. It is desirable, therefore, as well as inevitable, that his intellect partake of domesticity. But though the medical man should have something of the woman about him, it is very undesirable that he be all woman, much less all "old woman." There are certain dangers and disabilities associated with this kind of domestic occupation; and some of these we propose to point out and warn against.

All minds require opposition for their development, and for the continuous putting forth of their best powers. The family doctor who is fairly established in the confidence and affection of his clients seldom meets even with questioning, much less with contradiction. His lady patients offer him the incense of their enthusiastic confidence, and their children reward his benevolence with their love. The doctor is happy in this state of things, and considers it the just reward of his diligent devotion to duty. And so it is. But exactly as the riches of the wealthy man and the too early fame of the poet may be their intellectual ruin, so may the atmosphere of domestic worship, in which the family physician often lives and moves, make him self-satisfied, feeble in intellect, and degenerate in manhood. Who, or what is to save the successful doctor from becoming a good-natured, successful, but brainless old fool? Nothing can save him unless he will save himself.

That what has been advanced is strictly in accordance with truth may be easily proved by one of the commonest facts of every day experience. If there were placed together in a judgment hall an average middle-aged doctor, a lawyer, a clergyman, a statesman, and a man of business, and if twenty different people were asked which of the five was likely to be the most infallible in his judgments, and could least brook opposition to his authority, nineteen of the twenty judges would promptly select the doctor. Now, that is worth a pause and a few minutes' consideration. Is the doctor really entitled to pronounce more authoritatively on medical questions than the lawyer is on law, the statesman on state policy, or the man of trade on business? Are the physiological, pathological, and therapeutic problems involved in the practice of medicine really easier of solution and more capable of demonstration than corresponding problems in law, statesmanship, and commerce? We will not ask the doctor to speak his answer aloud so that all the world may hear; he shall utter it privately, in the softest of whispers, into the sympathetic ears of his professional brethren. What are the words of that whispered answer? These! "The most difficult, the most doubtful, and the most incapable of clear demonstration of all the practical problems that men are compelled by duty to deal with, are those connected with medical diagnosis and treatment."

But if that be the innermost conviction of every honest and intelligent doctor's mind, why such airs of professional infallibility, such assumptions of unquestionable authority? The medical man who has reached this stage constantly stands upon a pyramid with the base uppermost. That pyramid must inevitably fall over from time to time, and plunge him, on the one hand into a pit out of which nothing but hard lying and false swearing can deliver him, or, on the other, into a quagmire from which he can only be extricated by the frankest confession of error or mistaken judgment. What satisfaction can it be to any man to be accounted infallible when in his secret heart he knows that in the nature of the case no approximation to infallibility is so much as possible for him? It may be argued that some medical men honestly think themselves incapable of falling into error. We cannot, however, bring ourselves to believe that even among the most ignorant of doctors, a single one can be found who is so hopelessly stupid as that.

A man of real mental capacity always strives to take a just measure both of himself and of the circumstances of his daily life. For such a man recognises that he is of a certain value. He does not regard himself as a cab-horse, to be whipped about and reined up at the driver's pleasure, and to spend his days in indefatigable trotting, until old age shall consign him to the knacker's yard. A man has, or ought to have, a mind all his own, over which no other man, or body of men, or circumstances of life should ever be permitted to exercise absolute control. The mind, like personal property, should be put out at interest, so that it may constantly improve, expand, and become more productive to the very last day of time existence. But what happens to many a doctor after middle age? He is never contradicted; he has long ceased to read; his mind is made up on every point both of diagnosis and of treatment; routine is not only his religion but his daily bread. Under these circumstances the inactive brain can do nothing but shrink more and more until practical senility is the distinguishing feature of the mental faculties, whilst the body remains comparatively robust. The really capable medical man does not fail to see this dangerous tendency of his peculiarly domestic calling, and to take effectual steps for its counteraction.

Many practitioners of medicine regard their daily work and their weekly medical journal as all-sufficient for mental stimulus, mental development, and relaxation. They consider that to be a doctor only is to be a man of science, of education, and of intellectual weight. This, in many cases, is sheer nonsense. Lord Clarendon, the historian, said of the ecclesiastics of his time—"The clergy, who know the least, and take the worst measure of human affairs of all mankind who can write and read." That was a hard saying; but who knows what peculiar experiences of clerical persons had led so stout a conservative as Clarendon to so very contemptuous a conclusion? We do not say that any such sentiments could properly be expressed of the medical men of the present day; but we do say that the average doctor who has no interest outside his daily work, who knows nothing of the correcting, illuminating, and stimulating value of general literature, who does not associate himself with the public affairs of town or country, and who gives himself up absolutely and exclusively to physic and to nothing else, is often intellectually of little more consequence than his own horse, and professionally may be compared with the routine phlebotomists and black-draught specialists of a recent but discreditable past.

Happily, alike for doctors and for patients, the unintellectual dullards of the novelist and the writer of satirical verse are fast disappearing from the medical profession. It will be a welcome day when the last of them shall be seen no more. Their miserable traditions still hang about us and trammel the freer intellect of the present generation. But those traditions, like ghosts and witches, will also disappear before the rising dawn of science and liberal culture. Medical men will not long be content to be the mere handmaids of sickness and physical disorder. They will recognise that human happiness and human development lie quite as much within the scope of their duty as care for the weak and the sick. The State, too, will come to understand, and not only the State but ordinary men and women, that doctors have much to teach as well as much to practice; and that it is as important for the healthy to walk by the light of the medical man's science as it is to be well governed, or to provide for the teaching of a reasonable religion. But in order that this more rational, and in every way manlier and more joyous time may arrive speedily, the doctor must recognise that he cannot continue to be a mere domestic convenience. He must be not only a man of science but also of liberal culture; a man of the world who knows the world by contact with it and with the best minds in it. He must learn to measure himself by men not of his own fraternity; and to measure the relative importance to mankind of his art and calling with that of agriculture, commerce, law, religion, and the other great interests of humanity. He must, in short, be not merely a doctor but a man.

